# A Cost-Effectiveness Analysis: First-Line Avelumab Plus Axitinib Versus Sunitinib for Advanced Renal-Cell Carcinoma 

**DOI:** 10.3389/fphar.2020.00619

**Published:** 2020-05-08

**Authors:** Peiyao Lu, Weiting Liang, Jiahao Li, Yanming Hong, Zhuojia Chen, Tao Liu, Pei Dong, Hongbing Huang, Tiantian Zhang, Jie Jiang

**Affiliations:** ^1^ College of Pharmacy, Jinan University, Guangzhou, China; ^2^ Sun Yat-sen University Cancer Center, State Key Laboratory of Oncology in South China, Collaborative Innovation Center for Cancer Medicine, Guangzhou, China; ^3^ International Cooperative Laboratory of Traditional Chinese Medicine Modernization and Innovative Drug Development of Chinese Ministry of Education (MOE), Jinan University, Guangzhou, China; ^4^ Dongguan Institute of Jinan University, Dongguan, China

**Keywords:** cost-effectiveness, avelumab, axitinib, renal cell carcinoma, programmed death receptor

## Abstract

**Background:**

Compared with the standard of care with sunitinib, avelumab plus axitinib can increase progression-free survival in the first-line of advanced renal cell carcinoma (RCC), but the economic effect of the treatment is unknown. The purpose of the research was to evaluate the cost-effectiveness of the avelumab plus axitinib versus sunitinib in first-line treatment for advanced RCC from the US payer perspective.

**Methods:**

A Markov model was developed to evaluate the economic and health outcomes of avelumab plus axitinib vs sunitinib in the first-line setting for advanced RCC. The clinical data were obtained from the JAVELIN Renal 101 Clinical Trials. Deterministic and probabilistic sensitivity analyses were performed to assess uncertainty in the model. Health outcomes were measured in quality-adjusted life-years (QALYs).

**Results:**

The incremental cost-effectiveness ratio (ICER) of avelumab plus axitinib compared with sunitinib was $565,232 per QALY, the costs were $884,626 and $669,838, QALYs were 3.67 and 3.29, respectively. Sensitivity analysis demonstrated that differences in utilities in PFS and after progression were the most influential factors within the model. When avelumab was at 30% of the full price or axitinib was at 40% of the full price, avelumab and axitinib were approved to be cost-effective if the WTP threshold was $150,000 per QALY. The subgroup analysis showed the ICER of avelumab plus axitinib compared with sunitinib for the patients with PD-L1–positive tumors was $588,105.

**Conclusion:**

Avelumab plus axitinib in the first-line treatment was not cost-effective in comparison with sunitinib when the threshold of willingness to pay (WTP) was $150,000 per QALY.

## Introduction

The United States has the highest incidence of kidney cancer in the world (an age-standardized rate of 12 per 100,000), with a cumulative risk of 1.8 percent for men and 0.9 percent for women (Capitanio et al., 2019). In the USA, 5-year relative survival for patients with RCC is 92.5%; however, it drops to 65.7% in patients with locally advanced RCC (Umeyama et al., 2017). There are estimated to be 400,000 new cases of RCC worldwide every year (Rassy et al., 2020). The Global Burden of Disease 2015 Study illustrated that kidney cancer accounted for 1.60% of disease burden and was ranked 18th around the world according to the cancer mortality data (Fitzmaurice et al., 2017).

Recently, immune checkpoint inhibitors (ICIs) which target inhibitory receptors on T cells and generate antitumor immune mechanisms gradually draw more attention to the oncotherapy area (Havel et al., 2019). Compared with other immunotherapy, programmed cell death 1 (PD-1) and its ligand, PD1 ligand 1 (PD-L1) demonstrated a good effect on durable tumor regression and stabilization of disease (Brahmer et al., 2012). There are six antibodies against PD-1 or PD-L1 approved by the United States Food and Drug Administration (FDA): nivolumab, pembrolizumab, atezolizumab, avelumab, durvalumab, and cemiplimab. FDA has approved nivolumab, pembrolizumab, avelumab as the first-line treatment for patients with advanced RCC (FDA, 2019c). And the drug combinations are nivolumab plus ipilimumab, pembrolizumab plus axitinib, and avelumab plus axitinib, respectively.

The JAVELIN Renal 101 trial showed patients with RCC in first-line treatment received a combination of avelumab plus axitinib had longer progression-free survival (PFS) and a higher objective response rate than those who received sunitinib. The JAVELIN Renal 101 was a phase 3 trial. 886 patients at 144 sites in 21 countries were assigned in the trial and the median age of patients was 61.0 years old (range:27.0–88.0) (Motzer et al., 2019).

Avelumab is an antibody against PD-L1 and become the first approved drug for Merkel cell carcinoma and Locally Advanced or Metastatic Urothelial Carcinoma. Axitinib is a selective inhibitor of VEGFRs 1–3 which recommended for patients with metastatic RCC according to National Comprehensive Cancer Network (NCCN) in 2019. Sunitinib is recommended for the standard of care by Current treatment guidelines for patients with mRCC in order to stop renal tumors growing (Motzer et al., 2007). However, there is no evidence that the obvious overall survival (OS) benefit is described (Powles et al., 2017).

As an immune checkpoint inhibitor, avelumab showed its potential to treat patients with RCC. However, whether the cost of this treatment shows reasonable value is a great concern of stakeholders of US healthcare system such as policymakers, healthcare payers and providers and patients. To our knowledge, there is no relevant economic analysis about avelumab plus axitinib for RCC in the United States, so we conducted this study to evaluate the cost-effectiveness of avelumab plus axitinib versus sunitinib in the first-line treatment for advanced RCC from the perspective of the US payer.

## Patients and Methods

### Patients and Intervention

Our research was based on the trial of patients with advanced RCC in first-line treatment, JAVELIN Renal 101 Clinical Trials. And we used the clinical data from the trial (Motzer et al., 2019).

According to the JAVELIN Renal 101 Clinical Trials, sunitinib was given orally at a dose of 50 mg/day for the first 4 weeks of each 6-week cycle. In the avelumab plus axitinib group, patients were given avelumab (10 mg per kilogram of body weight) intravenously every 2 weeks plus axitinib (5 mg) orally twice daily. Due to the average of the body weight in American is 74.7 Kg, we assumed the body weight is 70 Kg for the weight loss effects in disease (Oh et al., 2017). Grade 3 or 4 adverse events (≧5%) were simultaneously modeled which included hypertension, diarrhea, anemia, thrombocytopenia, neutropenia, palmar–plantar erythrodysesthesia syndrome.

As shown in the JAVELIN Renal 101 Clinical Trials, 20.8% of patients (92 of 442) in the avelumab plus axitinib group and 39.2% of patients (174 of 444) in the sunitinib group received subsequent therapy after discontinued first-line treatment. The most common subsequent treatments in avelumab plus axitinib group were cabozantinib (30.4%); while in the sunitinib group were cabozantinib (10.8%), sunitinib (8.9%), and nivolumab (41.4%). The drug dose information was derived from labels of a drug reported by FDA, which was as followed: nivolumab 240 mg every 2 weeks (FDA, 2019b), cabozantinib 60 mg once daily (FDA, 2019a), sunitinib 50 mg once daily. We assumed that patients who did not receive subsequent therapy received supportive care.

### Decision-Analytic Markov Model

**Figure 1:**
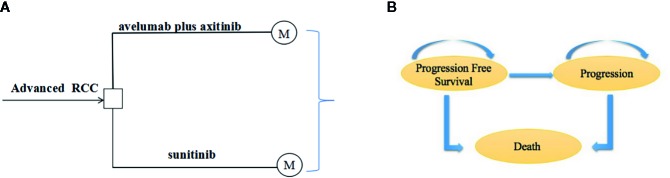
**(A)** State-transition for Markov model. **(B)** Influence diagram shows a network of three health states linked by transitional variables.

We developed a Markov model using TreeAge Pro 2018 (TreeAge, Williamstown, Massachusetts). The economic evaluation reporting followed the Consolidated Health Economic Evaluation Reporting Standards Statement (CHEERS) (Husereau et al., 2013) (**Supplementary Table S1**). The model structure was showed in **Supplementary Figure S1**. The model was constructed to evaluate the cost-effectiveness between avelumab plus axitinib and sunitinib. Half-cycle correction was applied for costs and health outcomes in the model. The three heath states in the model were progression-free survival, disease progression, and death (**Figures 1A, B**). The initial health state for all the patients was progression-free survival (Abdel-Rahman, 2017). Patients in the progression-free state were treated with avelumab plus axitinib or sunitinib until progression or death. After progression, patients were treated with subsequent anticancer drug therapies based on the JAVELIN Renal 101 Clinical Trials (Motzer et al., 2019).

The primary outputs of the model included total cost, quality-adjusted life years (QALYs), and incremental cost-effectiveness ratios (ICERs). Each model cycle represented 6 weeks, and the time horizon was 10 years because the Markov cohort analysis showed more than 90% of the patients will enter the death state in the 10^th^ year. Only direct medical care costs were included. The threshold of WTP we used was $150,000 (Sarfaty et al., 2018b). One-way sensitivity analysis was conducted to test the effects of parameter uncertainty of the model. Probabilistic sensitivity with 1,000 Monte Carlo simulations demonstrated the random effects of parameters.

### Transition Probabilities

We acquired the transition probabilities based on the method developed by Motzer et al. (2019). Data points were derived from Kaplan-Meier curves reported in the JAVELIN Renal 101 Clinical Trials by using R software, and then fitted to parametric survival models (log-logistic, Weibull, log-normal, and logistic). Weibull models were fitted to survival curves best according to the Akaike information criterion described as R-Square statistic. The probability for the transition from PFS to PD and any state to death was based on the following formulation: 

$$1-\exp\left[\lambda {\left(t-1\right)}^{\gamma }-\lambda t^{\gamma }\right]$$

t represents the cycle number in the Markov model (Diaby et al., 2014).

The parameters of Weibull models are shown in **Table 1**. Transition probabilities of different ages mortality rate was based on data from American life tables (Arias and Xu, 2018). We chose the starting age at 61 years according to the baseline characteristics of patients reported by the JAVELIN Renal 101 Clinical Trials.

**Table 1:** Key clinical and health preference data.

Parameters	Values	Distribution	Reference
Weibull survival model of PFS of avelumab plus axitinib	Scale = 0.05483; Shape = 0.97914; r2 = 0.97		
Weibull survival model of PFS of sunitinib	Scale = 0.07396; Shape = 1.02298; r2 = 0.98		
Weibull survival model of OS of avelumab plus axitinib	Scale = 0.00821; Shape = 1.16584; r2 = 0.99		
Weibull survival model of OS of sunitinib	Scale = 0.01117; Shape = 1.11967; r2 = 0.99		
Probability (%) of hypertension (grade≧3)		β	(Motzer et al., 2019)
avelumab plus axitinib	25.6		
sunitinib	11.7		
Probability (%) of diarrhea (grade≧3)		β	(Motzer et al., 2019)
avelumab plus axitinib	6.7		
sunitinib	–		
Probability (%) of anemia (grade≧3)		β	(Motzer et al., 2019)
avelumab plus axitinib	–		
sunitinib	8.2		
Probability (%) of thrombocytopenia (grade≧3)		β	(Motzer et al., 2019)
avelumab plus axitinib	–		
sunitinib	6.2		
Probability (%) of Neutropenia(grade≧3)		β	(Motzer et al., 2019)
avelumab plus axitinib	–		
sunitinib	8		
Probability (%) of palmar–plantar erythrodysesthesia syndrome (grade≧3)		β	(Motzer et al., 2019)
avelumab plus axitinib	5.8		
sunitinib	–		
Health utilities			
Utility of PFS			
avelumab plus axitinib	0.82	β	(Wan et al., 2019)
sunitinib	0.73	β	(Wan et al., 2019)
Utility of PD			
avelumab plus axitinib	0.66	β	(Wan et al., 2019)
sunitinib	0.66	β	(Wan et al., 2019)
Price of avelumab per 10mg	81.742	γ	(Services, 2019)
Price of axitinib per 5mg	213.154 (Range: 170.52–255.78)	γ	(Wu et al., 2018)
Price of sunitinib per 50mg	623.08 (Range: 498.46–747.7)	γ	(Wu et al., 2018)
Price of Nivolumab per mg	27.498 (Range: 22–33)	γ	(Wu et al., 2018)
Price of Cabozantinib per 60mg	491.299 (Range: 393.04–589.56)	γ	(Wu et al., 2018)
Cost of administration per unit	302.27 (Range: 241.82–362.73)	γ	(Wu et al., 2018)
Cost of supportive care	1256 (Range: 1,022–1,489)	γ	(Wu et al., 2018)
Cost of adverse events (grade≧3) per event			
Hypertension	209.004 (Range: 167.20–250.81)	γ	(Wu et al., 2018)
Diarrhea	5,991.38 (Range: 4,793.104–7,189.656)	γ	(Perrin et al., 2015)
Anemia per month	1,947.189 (Range: 1,557.751–2,336.627)	γ	(Sarfaty et al., 2018b)
Thrombocytopenia	4,155.245 (Range: 3,324.20–4,986.29)	γ	(Wu et al., 2018)
Neutropenia	1,060.986 (Range: 848.79–1,273.18)	γ	(Georgieva et al., 2018)
palmar–plantar erythrodysesthesia syndrome	122.98 (Range: 3.43–1748)	γ	(Wu et al., 2018)

AE, adverse event; PD, progressed disease; PFS, progression-free survival; OS, overall survival.

### Costs and Utilities

Direct medical costs were considered including drug, administration, and adverse event (AE) costs. Original costs of avelumab were acquired from average sale price from the U.S. Centers for Medicare & Medicaid Services (2019). Cost of axitinib and sunitinib and drugs in subsequent anticancer drug therapies were referred from the previous research (Wu et al., 2018). These costs are shown in **Table 1**. Cost of administration and cost of supportive care were obtained from Wu et al. (2018). Costs of grade 3 or 4 adverse events and subsequent anticancer drug therapies were accessed from the published research (Wu et al., 2018) (Perrin et al., 2015) (Sarfaty et al., 2018a) (Georgieva et al., 2018) (**Table 1**). We discounted all costs and health outcomes at a 3% annual rate for the inflation adjustment and all costs were inflated to 2019 US dollars by the US consumer price index (Economic Research at the St. Louis Fed, 2019).

QALYs was obtained by combining the two dimensions of survival time and health-related quality of life (QOL) which is generally regarded as utility (health status value, from 0 to 1 for death to 1 for perfect health). Health state utilities were referred to the previously published cost-effectiveness analyses of the drug to treat metastatic RCC (Wan et al., 2019).

### Sensitivity Analysis

The one-way sensitivity analysis was performed to evaluate the influence of parameter uncertainty in the model. We tested the effect of each parameter separately on ICERs for variables. Utilities were referenced in the same disease from American data (Wan et al., 2019). The ranges of the parameters used in the one-way sensitivity analyses were acquired from the published article; if data were not available, ± 20% of the base-case value was used in range (Sarfaty et al., 2018b).

Probabilistic sensitivity analyses (PSA) were also conducted to illustrate the robustness of our analysis results. γ distribution was assumed for costs, β distribution was for utility values and probabilities (Fragoulakis et al., 2013) (Usmani et al., 2016). We performed 1,000 Monte Carlo simulations, each time stochastically sampling from the distributions of all parameters. Cost-effectiveness curves were designed to figure out which scheme is more cost-effective. A WTP threshold of $150,000 per QALY gained was used for the analysis.

Subgroup analysis was also conducted in patients with PD-L1–positive tumors. PFS hazard ratio (HR) for the subgroup was used.

## Results

### Model Validation

Weibull distribution was proved to fit survival curves calculated by evaluation criteria and visual inspection (**Supplementary Figure S2**). The goodness-of-fit test demonstrated that the adjusted R^2^ values for the model-generated PFS of avelumab plus axitinib and sunitinib were 0.97 and 0.98, OS of avelumab plus axitinib and sunitinib were 0.99 and 0.99 (**Table 1**).

### Base Case Analysis

The base-case cost effectiveness of avelumab plus axitinib versus sunitinib were shown in **Table 2**. For mRCC patients, the costs of avelumab plus axitinib and sunitinib were $884,626 and $669,838, QALYs of 3.67 and 3.29, respectively. The ICER of avelumab plus axitinib compared with sunitinib was $565,232 per QALY (**Table 2**).

**Table 2:** Results for base case and subgroup analysis.

	Avelumab	Axitinib	Costs ($)			QALYs			ICER($/QALY)
			Sunitinib	Avelumab plus Axitinib	Incremental Costs	Sunitinib	Avelumab plus Axitinib	Incremental QALY	
**Overall Population**	**Avelumab and Axitinib at full price**		669,838	884,626	214,788	3.29	3.67	0.38	565,232
	**Avelumab at 30% of full price and Axitinib at full price**		669,838	702,871	33,033	3.29	3.67	0.38	86,929
	**Avelumab at full price and Axitinib at 40% of full price**		669,838	722,128	52,290	3.29	3.67	0.38	137,605
**Patients with PD-L1–Positive Tumors**	**Avelumab and Axitinib at full price**		668,899	904,141	235,242	3.29	3.69	0.40	588,105

QALY, quality-adjusted life-year; ICER, incremental cost-effectiveness ratio; PD-1, programmed cell death 1; PD-L1, PD1 ligand 1.

### Sensitivity Analysis

**Figure 2:**
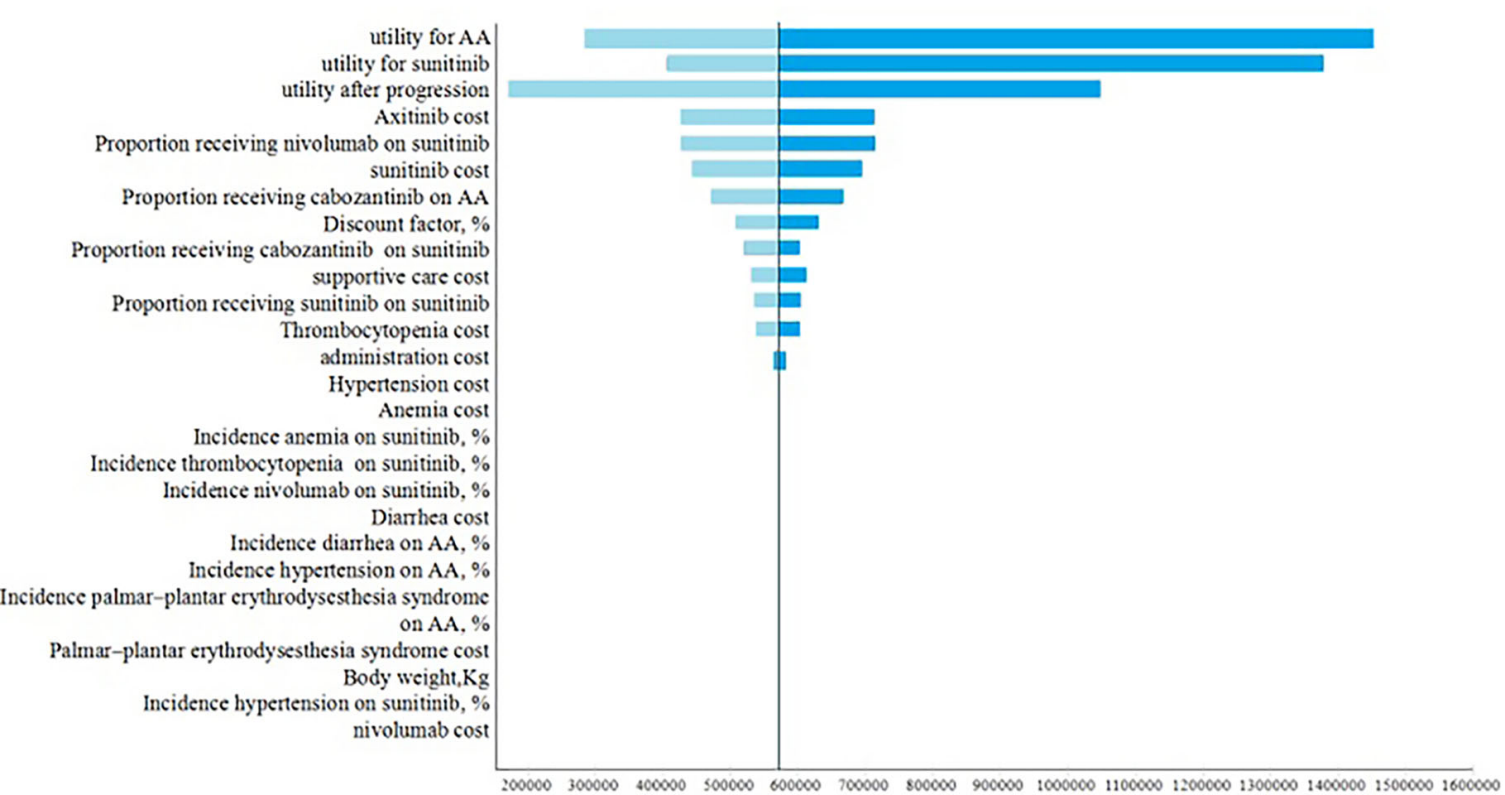
The results of univariable sensitivity analysis.

One-way sensitivity analyses demonstrated utilities in PFS and after progression were the most influential factors within the model (**Figure 2**). Other variables, such as the drug costs, cost of adverse events and proportions of receiving subsequent therapy, had a moderate or minor influence on the ICER.

**Figure 3:**
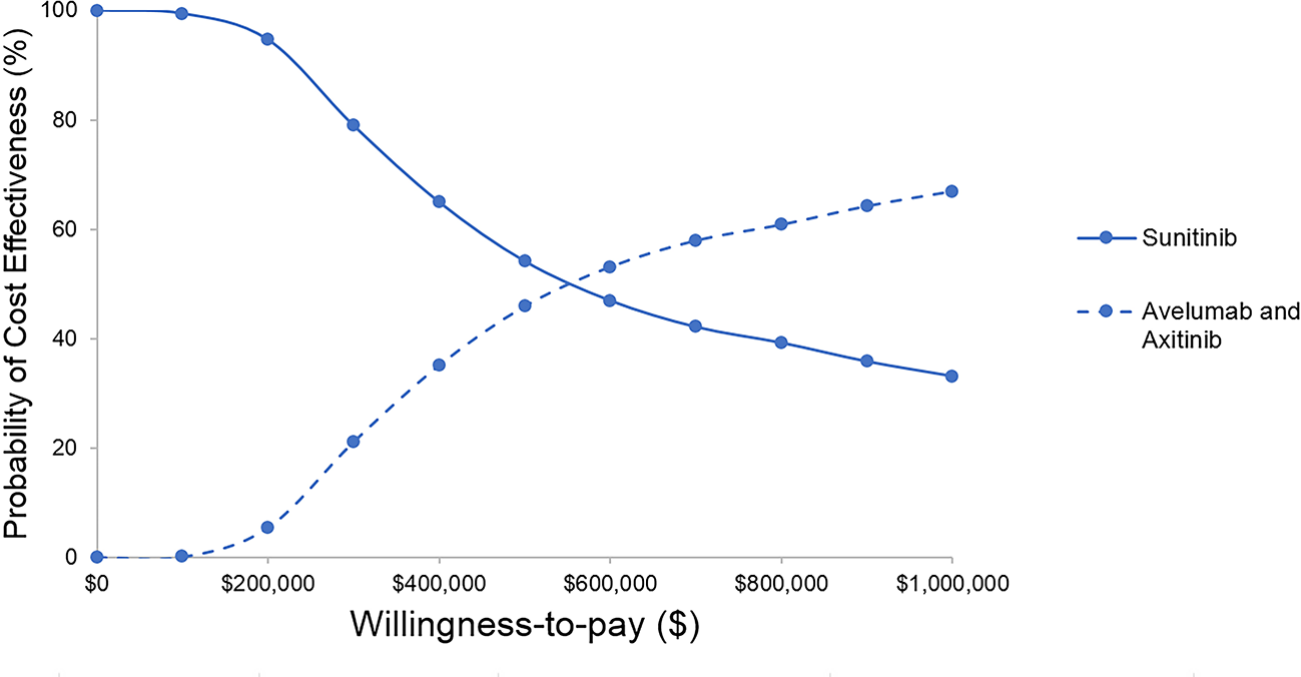
Cost-effectiveness acceptability curve.

The results of probabilistic sensitivity analyses demonstrated that the probability of avelumab plus axitinib being cost-effective compared with sunitinib is 2% at a willingness-to-pay threshold of $150,000 per QALY (**Figure 3**). When avelumab was at 30% of the full price or axitinib was at 40% of the full price, the ICER was $86,929, $137,605, respectively (**Table 2**).

The result of subgroup analysis showed that avelumab plus axitinib was not cost-effective and the ICER of avelumab plus axitinib compared with sunitinib for the patients with PD-L1–positive tumors was $588,105 (**Table 2**).

## Discussion

Our research was the first study to evaluate the cost-effectiveness of avelumab plus axitinib versus sunitinib. The combination of avelumab plus axitinib showed better efficacy than sunitinib; however, it was not cost-effective when avelumab and axitinib are at the current price. Avelumab plus axitinib cost $565,232 per additional QALY gained compared with sunitinib. The probabilistic sensitivity analyses showed it was only 2% that avelumab plus axitinib would be cost-effective when the WTP threshold was $150,000 per QALY. Based on this case, proposals for price reduction were provided to solve the situation. When avelumab was at 30% of the full price or axitinib was at 40% of the full price, avelumab and axitinib were approved to be cost-effective if the WTP threshold was $150,000 per QALY. One-way sensitivity analysis showed the utilities of PFS and after progression had the greatest influence on the ICER. Subgroup analysis showed that avelumab plus axitinib was not cost-effective and the ICER of avelumab plus axitinib compared with sunitinib for the patients with PD-L1–positive tumors was $588,105.

A recent study compared avelumab with chemotherapy, standard care, and best supportive care for UK metastatic merkel cell carcinoma (mMCC) patients and found out that avelumub was cost-effective as the WTP threshold of £50,000 per QALY (Bullement et al., 2019). There are some reasons why a different conclusion was provided. First, mMCC patients were treated by a single drug avelumab without axitinib while advanced RCC patients were treated by combination therapy of avelumab and axitinib. Second, different baseline drugs were used. Our research used sunitinib as a baseline drug and the article for mMCC used chemotherapy, standard care, best supportive care as baseline drugs. Third, different regions were based. Our research was based on the US payer perspective and the article for mMCC was based on the UK National Health Service perspective.

Due to the high costs in avelumab plus axitinib for mRCC patients, it is important to explore what the function of axitinib is and whether avelumab synergizes with axitinib. It is noticeable that axitinib showed great clinical activity in the first-line setting of mRCC compared with placebo (Rini et al., 2013). Monotherapy about avelumab or axitinib should be further investigated as more cost-effective strategies.

There are some published studies about cost-effectiveness analysis of other PD-1/PD-L1 in RCC patients. The cost-effectiveness analysis of nivolumab plus ipilimumab versus sunitinib for the patient with mRCC showed nivolumab plus ipilimumab was cost-effective with an ICER of $108,363 per QALY gained in United States (Wan et al., 2019). A similar economic evaluation comparing nivolumab plus ipilimumab to sunitinib indicated nivolumab plus ipilimumab was cost-effective in the United States or China, not in the United Kingdom (Wu et al., 2018). A cost-effectiveness analysis of nivolumab and ipilimumab versus sunitinib in first-line intermediate- to poor-risk advanced renal cell carcinoma showed nivolumab and ipilimumab was estimated to be cost-effective from the US payer perspective (Reinhorn et al., 2019). It seems nivolumab is more cost-effective than avelumab when treating for RCC patients. The cost-effectiveness analysis of pembrolizumab plus axitinib versus sunitinib in first-line advanced renal cell carcinoma in China showed pembrolizumab plus axitinib was not cost-effective at a threshold value of $29,306 per QALY (Chen et al., 2019). The ICER for pembrolizumab plus axitinib was $55,185 per QALY versus sunitinib.

The JAVELIN Renal 101 trial used sunitinib as the competitor, which was administered at the approved standard dosing schedule of 50 mg/day for 4 weeks followed by 2 weeks off. One recently published study showed that the alternative schedule (2-weeks on with 1-week break) might be more clinically beneficial to patients with RCC than the approved standard dosing schedule (Abogunrin et al., 2019), leading to an even greater ICER for avelumab plus axitinib strategy.

Our study has several limitations. First, the exact clinical data was not obtained, so the transition probabilities were fitted by parametric survival models. It could not adequately reflect real-world conditions, but our models matched well. Second, the utilities and costs were referred to as some previous studies which were about mRCC (Wu et al., 2018; Wan et al., 2019). However, the ranges of the utilities and these costs used in the sensitivity analysis account for the variation. Third, based on the randomized controlled trial data, our model cannot reflect real-world clinical situations, researches about real-world data should be further conducted. Forth, pazopanib and cabozantinib are other first-line treatments recommended by NCCN guidelines for kidney cancer, however, the head-to-head researches about avelumab plus axitinib versus pazopanib or cabozantinib have not been published yet. Fifth, the OS curve of the JAVELIN Renal 101 Clinical Trials was immature, further research should be conducted as soon as the updated data could be accessible. Finally, we used the published data to simulate long-term benefit, which is likely to lead to biases. The long-term projection should be validated by real-world long-term observational data.

## Conclusions

Avelumab plus axitinib in the first-line treatment was not cost-effective in comparison with sunitinib when the threshold of willingness to pay (WTP) was $150,000 per QALY. When avelumab was at 30% of the full price or axitinib was at 40% of the full price, avelumab, and axitinib were approved to be cost-effective if the WTP threshold was $150,000 per QALY.

## Data Availability Statement

All datasets generated for this study are included in the article/**Supplementary Material**.

## Author Contributions

PL and WL developed the economic model and performed the analyses. JL and TZ interpreted the results and wrote the draft manuscript. PL, TZ, YH, ZC, PD, JL, and TL reviewed, analyzed, and interpreted the data. TZ, HH, and JJ contributed to the design of the primary model and the interpretation of the results. All authors reviewed and approved the final version.

## Funding 

This study was supported by the National Natural Science Foundation of China (grant no.71704064) and the Natural Science Foundation of Guangdong Province, China (grant no. 2017A030310174).

The funders had no role in the design and conduct of the study; collection, management, analysis, and interpretation of the data; preparation, review, or approval of the manuscript; and decision to submit the manuscript for publication. There are no non-monetary sources of support.

## Conflict of Interest

The authors declare that the research was conducted in the absence of any commercial or financial relationships that could be construed as a potential conflict of interest.
