# Supplementary material for: A Cost-Effectiveness Analysis: First-Line Avelumab Plus Axitinib Versus Sunitinib for Advanced Renal-Cell Carcinoma
Source: Front Pharmacol. 2020 May 8;11:619. doi: 10.3389/fphar.2020.00619 (PMC7225300; doi:10.3389/fphar.2020.00619)
Supplement: Supplementary file 2 [file DataSheet_2.docx]

**Supplemental Material**

**Title:A Cost-effectiveness Analysis: First-line Avelumab plus Axitinib vs Sunitinib for Advanced Renal-Cell Carcinoma**

**Running title: Cost-effectiveness of Avelumab for RCC**

Peiyao Lu, MSc1 †, Weiting Liang, MSc2,3,4,5†, Jiahao Li, MSc1, Yanming Hong, MSc1, Zhuojia Chen, PhD6, Tao Liu, MSc 6, Pei Dong, MD6, Hongbing Huang, MSc6*, Tiantian Zhang, PhD1,7*, Jie Jiang, PhD1,7,8*

1 College of Pharmacy, Jinan University, Guangzhou 510632, China

2 Department of Pharmacy, Sun Yat-sen University Cancer Center, Guangzhou 510060, China

3 State Key Laboratory of Oncology in South China, Sun Yat-sen University, Guangzhou 510060, China

4 Collaborative Innovation Center for Cancer Medicine, Sun Yat-sen University, Guangzhou 510060, China

5 Guangdong Key Laboratory of Nasopharyngeal Carcinoma Diagnosis and Therapy, Sun Yat-sen University, Guangzhou 510060, China

6 Sun Yat-sen University Cancer Center; State Key Laboratory of Oncology in South China; Collaborative Innovation Center for Cancer Medicine，Guangzhou 510060, China

7 International Cooperative Laboratory of Traditional Chinese Medicine Modernization and Innovative Drug Development of Chinese Ministry of Education (MOE), Jinan University, Guangzhou 510632, China

8 Dongguan Institute of Jinan University, China

† These authors have contributed equally to this work.

*** Correspondence:**

Jie Jiang

jiangjie218@126.com

Tiantian Zhang

ztt_84@126.com

Hongbing Huang

huanghb@sysucc.org.cn


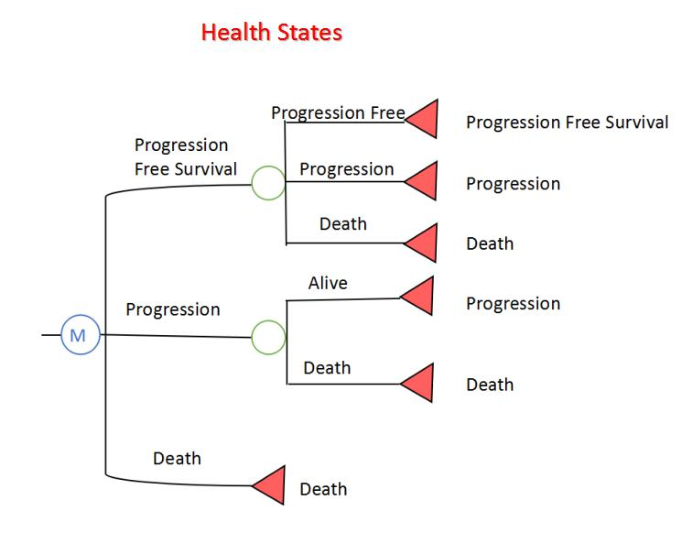


Figure 1 in the Supplement. Structure for Markov model.


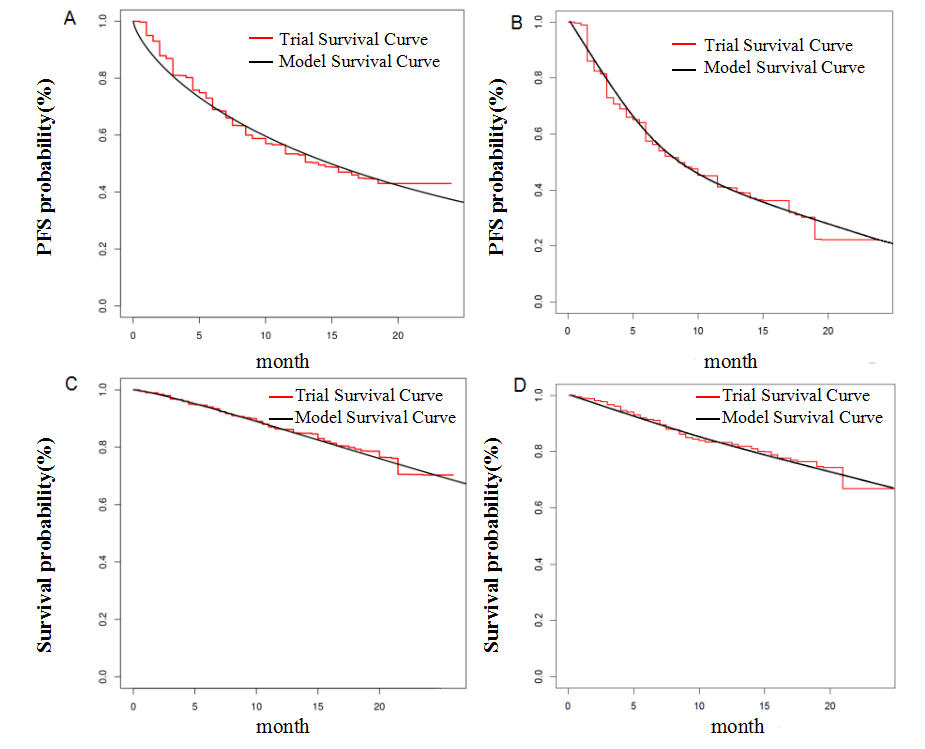


Figure 2 in Supplement. Cost-effectiveness model validation results. A shows the PFS survival curve of avelumab plus axitinib. B shows the PFS survival curve of sunitinib. C shows the OS curve of avelumab plus axitinib. D shows the OS curve of sunitinib.

PFS, progression-free survival; OS, overall survival
